# Impact of low-level viremia with drug resistance on CD4 cell counts among people living with HIV on antiretroviral treatment in China

**DOI:** 10.1186/s12879-022-07417-z

**Published:** 2022-05-04

**Authors:** Pengtao Liu, Yinghui You, Lingjie Liao, Yi Feng, Yiming Shao, Hui Xing, Guanghua Lan, Jianjun Li, Yuhua Ruan, Dan Li

**Affiliations:** 1 Weifang Medical University, Weifang, People’s Republic of China; 2 State Key Laboratory for Infectious Disease Prevention and Control (SKLID), National Center for AIDS/STD Control and Prevention (NCAIDS), Chinese Center for Disease Control and Prevention (China CDC), Collaborative Innovation Center for Diagnosis and Treatment of Infectious Diseases, 155 Changbai Road, Changping District, Beijing, 102206 People’s Republic of China; 3 Guangxi Key Laboratory of Major Infectious Disease Prevention Control and Biosafety Emergency Response, Guangxi Center for Disease Control and Prevention, Nanning, People’s Republic of China

**Keywords:** HIV, Low-level viremia, HIV drug resistance, Drug resistance associated mutations, China

## Abstract

**Background:**

Maintaining plasma HIV RNA suppression below the limit of quantification is the goal of antiretroviral therapy (ART). When viral loads (VL) remain in low-level viremia (LLV), or between 201 and 999 copies/mL, the clinical consequences are still not clear. We investigated the occurrence of LLV with drug resistance and its effect on CD4 cell counts in a large Chinese cohort.

**Methods:**

We analysed data of 6,530 ART-experienced patients (42.1 ± 10.9 years; 37.3% female) from the China’s national HIV drug resistance (HIVDR) surveillance database. Participants were followed up for 32.9 (IQR 16.7–50.5) months. LLV was defined as the occurrence of at least one viral load (VL) measurement of 50–200 copies/mL during ART. Outcomes were drug resistance associated mutations (DRAM) and CD4 cell counts levels.

**Results:**

Among 6530 patients, 58.0% patients achieved VL less than 50 copies/mL, 27.8% with VL between 50 and 999 copies/mL (8.6% experienced LLV), and 14.2% had a VL ≥ 1000 copies/mL. Of 1818 patients with VL 50–999 copies/mL, 182 (10.0%) experienced HIVDR, the most common DRAM were M184I/V 28.6%, K103N 19.2%, and V181C/I/V 10.4% (multidrug resistance: 27.5%), and patients with HIVDR had a higher risk of CD4 cell counts < 200 cells/*μL* (AOR 3.8, 95% CI 2.6–5.5, p < 0.01) comparing with those without HIVDR. Of 925 patients with VL ≥ 1000 copies/mL, 495 (53.5%) acquired HIVDR, the most common DRAM were K103N 43.8%, M184I/V 43.2%, M41L 19.0%, D67N/G 16.4%, V181C/I/V 14.5%, G190A/S 13.9% and K101E 13.7% (multidrug resistance: 75.8%), and patients with HIVDR had a higher risk of CD4 cell counts < 200 cells/*μL* (AOR 5.8, 95% CI 4.6–7.4, p < 0.01) comparing with those without HIVDR.

**Conclusion:**

Persistent with VL 50–999 copies/mL on ART is associated with emerging DRAM for all drug classes, and patients in this setting were at increased risk of CD4 cell counts < 200 cells/*μL*, which suggest resistance monitoring and ART optimization be earlier considered.

## Background

The use of antiretroviral therapy (ART) has resulted in substantial reductions in HIV/AIDS-related morbidity and mortality worldwide [1–4]. Maintaining plasma HIV RNA suppression below the limit of quantification is the goal of ART, which induces persistent suppression of HIV replication and gradual recovery of CD4 cell counts [3–5]. Durable viral suppression is accomplished with sustained ART adherence in the majority of people living with HIV (PLWH). However, some PLWH develop persistent low-level viremia (LLV), which is usually defined as plasma viral load (VL) between 50 and 200 copies/mL [1, 6–8]. It’s not an uncommon finding in clinical practice, with estimates of 30% of PLWH on ART experiencing LLV [9]. It should be noted that widely followed ART guidelines diverge in their interpretation and recommended management of persistent viremia of low magnitude, reflecting the limited evidence base for these common clinical findings [1, 10, 11].

During ART, PLWH may experience small increases in VL (50–200 copies/mL) that do not reach the threshold for virological failure (VF), known as LLV [1, 10, 11]. In developed countries with easy access to VL monitoring, LLV can be a concern for both patients and physicians [12–14]. In these countries, upon detection of raised VL higher than 50 copies/mL, interventions such as resistance testing, pharmacokinetic measurement, switch of ART regimen, and adherence counselling might already be initiated [10, 15]. Studies from developed countries have demonstrated that HIV-1 drug resistance (HIVDR) testing at a plasma VL < 1000 copies/mL provides potentially clinically useful information [10, 16, 17]. They have found that patients with LLV harbor drug resistance associated mutations (DRAM) that confer resistance to the current ART regimens and decreases future therapeutic options [10, 18]. However, the available evidence might not apply to ART programmes in China, where current National Free Antiretroviral Treatment Program (NFATP) guidelines recommend that annual VL testing and interventions are only advised if VL ≥ 1000 copies/mL [19–21]. Furthermore, available studies from China performed integrase genotyping only on samples with VL ≥ 1000 copies/mL, few data are available on the emergence of such mutations in patients with LLV [22–25]. In recent years, in-house resistance assays can be performed on samples with VL below 1000 copies/mL, and the improvement of assays to quantify VL has led to progressively decrease the threshold of VF [7, 21, 26, 27]. The high threshold for VF currently used in NFATP should be reconsidered.

To date, the implications and clinical significance of LLV with drug resistance are still not clear in China. Additionally, few or no studies have analyzed the possible role of LLV with drug resistance as a tool to predict CD4 cell count < 200 cells/*μL*, while patients who initiate ART with a CD4 > 200 cells/*μL* are at reduced risk of death and serious opportunistic infections [28–32]. Whether VF thresholds should be lowered in patients from NFATP is yet to be determined. Therefore, we conducted the present study to examine the effect of LLV with drug resistance on patients’ clinical outcomes in NFATP.

## Methods

### Participants and definitions

We assessed all the data from China’s national HIVDR surveillance database from Jan 1, 2008, to Dec 31, 2015 (data downloaded on Dec 31, 2017). In this study, eligibility criteria were: 18 years or older; having received ART for more than 12 months; attending a participating clinic for routine HIVDR survey; having detailed viral load measurement data and CD4 cell count. In samples with VL ≥ 50 copies/mL, HIVDR genotyping was performed by in-house PCR as previously described [11, 23, 33]. HIVDR mutation analysis and viral subtype determination were performed on a 1.3 kb section of the HIV pol gene using the Stanford University HIVDR Database online sequence analysis tool: Genotypic Resistance Interpretation Algorithm (https://hivdb.stanford.edu/hivdb/by-mutations/, accessed Oct 16, 2018). NFATP guideline defines virological failure (VF) as a confirmed HIV RNA ≥ 1000 copies/mL. LLV was defined as the occurrence of at least one viral load (VL) measurement of 50–200 copies/mL during ART. The evaluation of CD4 cell count was performed after the event of LLV. The adherence questionnaire form included: (a) have you ever missed ARV drugs? (b) Have you ever missed ARV drugs because of side effects? (c) Have you ever missed ART because of excessive drugs? (d) Have you ever had difficulties taking ARV drugs at the exact time? For each question, a Likert scale: (a) never, (b) rarely, (c) sometimes, and (d) often is adopted in response. Never missing ARV drugs (or > 95% in multiple follow-up) is considered to be good adherence. Ethical approval for the study was obtained from the National Center for AIDS/STD Control and Prevention, China CDC Institutional Review Board. All individuals in this study provided written consent at the time of participation, and written informed consent was obtained from all study participants. All methods were performed in accordance with the relevant guidelines and regulations.

### Statistical analysis

The significant differences in categorical variables were analyzed using χ^2^ test or Fisher’s exact tests. Logistic regression was used to examine the associations between LLV, DR and CD4 cell counts. Weighted analyses were used to derive representative estimates of the detection rates of patients harboring HIV with mutations conferring resistance to ARV drugs belonging to the non-nucleoside reverse transcriptase inhibitor (NNRTI), transcriptase inhibitor (NRTI) and protease inhibitor (PI) classes. Weight was calculated based on the number of patients followed in each local center. All tests of significance were two-sided, with p < 0.05 indicating that an association was statistically significant. All the statistical analyses were performed with SAS 9.4 software (SAS Institute, Cary, NC, USA).

## Results

### Demographic characteristics of the study population

**Fig. 1:**
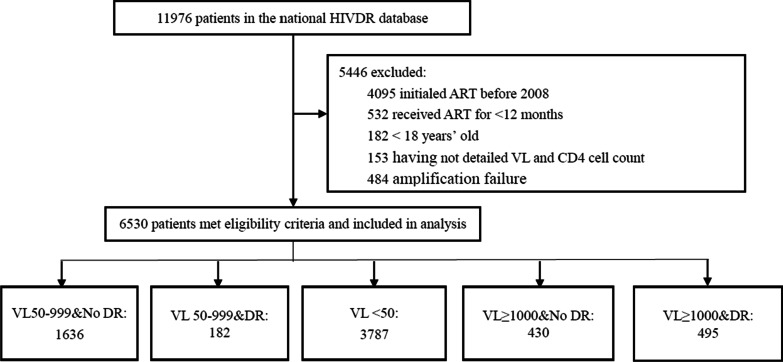
Study profile. 11976 patients were eligible in this study; 5446 did not meet eligibility criteria, and finally 6530 patients were included for analysis

11,976 PLWH were eligible in this study; 5446 did not meet eligibility criteria, and finally 6530 PLWH were included for analysis (Fig. 1). Of all the participants, 62.7% were male, 52.3% over 40 years old, 78.6% with an education level of secondary school or less, 60.4% farmers, 74.6% married, 40.5% infected through sexual intercourse, 12.8% with CD4 cell counts < 200 cells/*μL*, 14.2% with a VL ≥ 1000 copies/mL. All patients received free ART treatment through the NFATP, and most patients used lamivudine (3TC) based regimens (initial: 79.8%, at survey: 78.0%). The percentage of patients who missed dose in the past month was 26.1%. For more detailed information, see Table 1.

**Table 1:** General characteristics of patients who initiated ART from 2008 to 2015

Factor	N (%)	VL 50–999 and No DR (%)	VL 50–999 and DR (%)	VL < 50(%)	VL ≥ 1000 and No DR (%)	VL ≥ 1000 and DR (%)	p
Total	6530 (100%)	1636 (100%)	182 (100%)	3787 (100%)	430 (100%)	495 (100%)	
Sex							
Male	4097 (62.7%)	941 (57.5%)	110 (60.4%)	2453 (64.8%)	284 (66.0%)	309 (62.4%)	< 0.01
Female	2433 (37.3%)	695 (42.5%)	72 (39.6%)	1334 (35.2%)	146 (34.0%)	186 (37.6%)	
Age							
Median years (IQR)	41 (34–49)	44 (37–51)	39 (34–46)	40 (33–48)	41 (34–39)	42 (35–50)	< 0.01
≤ 30	940 (14.4%)	131 (8.0%)	29 (15.9%)	665 (17.6%)	56 (13.0%)	59 (11.9%)	
31–40	2174 (33.3%)	474 (29.0%)	70 (38.5%)	1305 (34.5%)	154 (35.8%)	171 (34.5%)	
> 40	3416 (52.3%)	1031 (63.0%)	83 (45.6%)	1817 (48.0%)	220 (51.2%)	265 (53.5%)	
Education							
Post-secondary school or more	1400 (21.4%)	229 (14.0%)	25 (13.7%)	1026 (27.1%)	70 (16.3%)	50 (10.1%)	< 0.01
Secondary school or less	5130 (78.6%)	1407 (86.0%)	157 (86.3%)	2761 (72.9%)	360 (83.7%)	445 (89.9%)	
Occupation							
Farmer	3943 (60.4%)	1197 (73.2%)	73 (40.1%)	2049 (54.1%)	259 (60.2%)	365 (73.7%)	< 0.01
Other	2552 (39.1%)	436 (26.7%)	108 (59.3%)	1709 (45.1%)	171 (39.8%)	128 (25.9%)	
Marital status							
Unmarried	1659 (25.4%)	326 (19.9%)	29 (15.9%)	1105 (29.2%)	103 (24.0%)	96 (19.4%)	< 0.01
Married	4871 (74.6%)	1310 (80.1%)	153 (84.1%)	2682 (70.8%)	327 (76.0%)	399 (80.6%)	
Route of HIV infection							
Sexual transmission	2647 (40.5%)	427 (26.1%)	78 (42.9%)	1917 (50.6%)	131 (30.5%)	94 (19.0%)	< 0.01
Other	3883 (59.5%)	1209 (73.9%)	104 (57.1%)	1870 (49.4%)	299 (69.5%)	401 (81.0%)	
Initial ART regimens							
D4T/3TC/EFV or NVP	2953 (45.2%)	600 (20.3%)	87 (2.9%)	1888 (63.9%)	199 (6.7%)	179 (6.1%)	< 0.01
AZT/3TC/EFV or NVP	1846 (28.3%)	667 (36.1%)	59 (3.2%)	889 (48.2%)	106 (5.7%)	125 (6.8%)	
TDF/3TC/EFV or NVP	555 (8.5%)	45 (8.1%)	1 (0.2%)	454 (81.8%)	39 (7.0%)	16 (2.9%)	
Other regimens	1176 (18.0%)	324 (27.6%)	35 (3.0%)	556 (47.3%)	86 (7.3%)	175 (14.9%)	
ART regimens at survey							
D4T/3TC/EFV or NVP	2760 (42.3%)	596 (21.6%)	70 (2.5%)	1795 (65.0%)	161 (5.8%)	138 (5.0%)	< 0.01
AZT/3TC/EFV or NVP	1321 (20.2%)	269 (20.4%)	59 (4.5%)	825 (62.5%)	75 (5.7%)	93 (7.0%)	
TDF/3TC/EFV or NVP	1014 (15.5%)	149 (14.7%)	17 (1.7%)	762 (75.1%)	51 (5.0%)	35 (3.5%)	
Other first-line regimens	125 (1.9%)	19 (15.2%)	2 (1.6%)	61 (48.8%)	19 (15.2%)	24 (19.2%)	
PI/r-based regimens	1310 (20.1%)	603 (46.0%)	34 (2.6%)	344 (26.3%)	124 (9.5%)	205 (15.6%)	
Duration of ART (months)							
Median months (IQR)	33 (17–50)	46 (23–61)	35 (24–50)	27 (15–48)	28 (17–49)	45 (22–57)	< 0.01
12–24	2576 (39.4%)	444 (27.1%)	45 (24.7%)	1748 (46.2%)	193 (44.9%)	146 (29.5%)	
> 24	3954 (60.6%)	1192 (72.9%)	137 (75.3%)	2039 (53.8%)	237 (55.1%)	349 (70.5%)	
CD4 cell counts (cells/*μL*) at survey							
Median cells/*μL* (IQR)	340 (234–443)	353 (256–452)	272 (189–351)	362 (260–460)	242 (157–328)	165 (69–264)	< 0.01
< 200	837 (12.8%)	195 (11.9%)	58 (31.9%)	244 (6.4%)	130 (30.2%)	210 (42.4%)	
≥ 200	5693 (87.2%)	1441 (88.1%)	124 (68.1%)	3543 (93.6%)	300 (69.8%)	285 (57.6%)	
HIV viral load (copies/mL) at survey							
Median log_10_ copies/mL (IQR)	1.3 (1.3–2.5)	2.2 (2.2–2.5)	1.7 (1.6–2.9)	1.3 (0.9–1.3)	4.3 (3.7–4.8)	4.4 (3.9–5.0)	< 0.01
< 1000	5605 (85.8%)	1636 (100.0%)	182 (100.0%)	3787 (100.0%)	0 (0.0%)	0 (0.0%)	
≥ 1000	925 (14.2%)	0 (0.0%)	0 (0.0%)	0 (0.0%)	430 (100.0%)	495 (100.0%)	
Missed doses in the past month							
No	4828 (73.9%)	993 (60.7%)	100 (54.9%)	3178 (83.9%)	318 (74.0%)	239 (48.3%)	< 0.01
Yes	1702 (26.1%)	643 (39.3%)	82 (45.1%)	609 (16.1%)	112 (26.0%)	256 (51.7%)	

### HIV-1 drug resistance and mutations

**Fig. 2:**
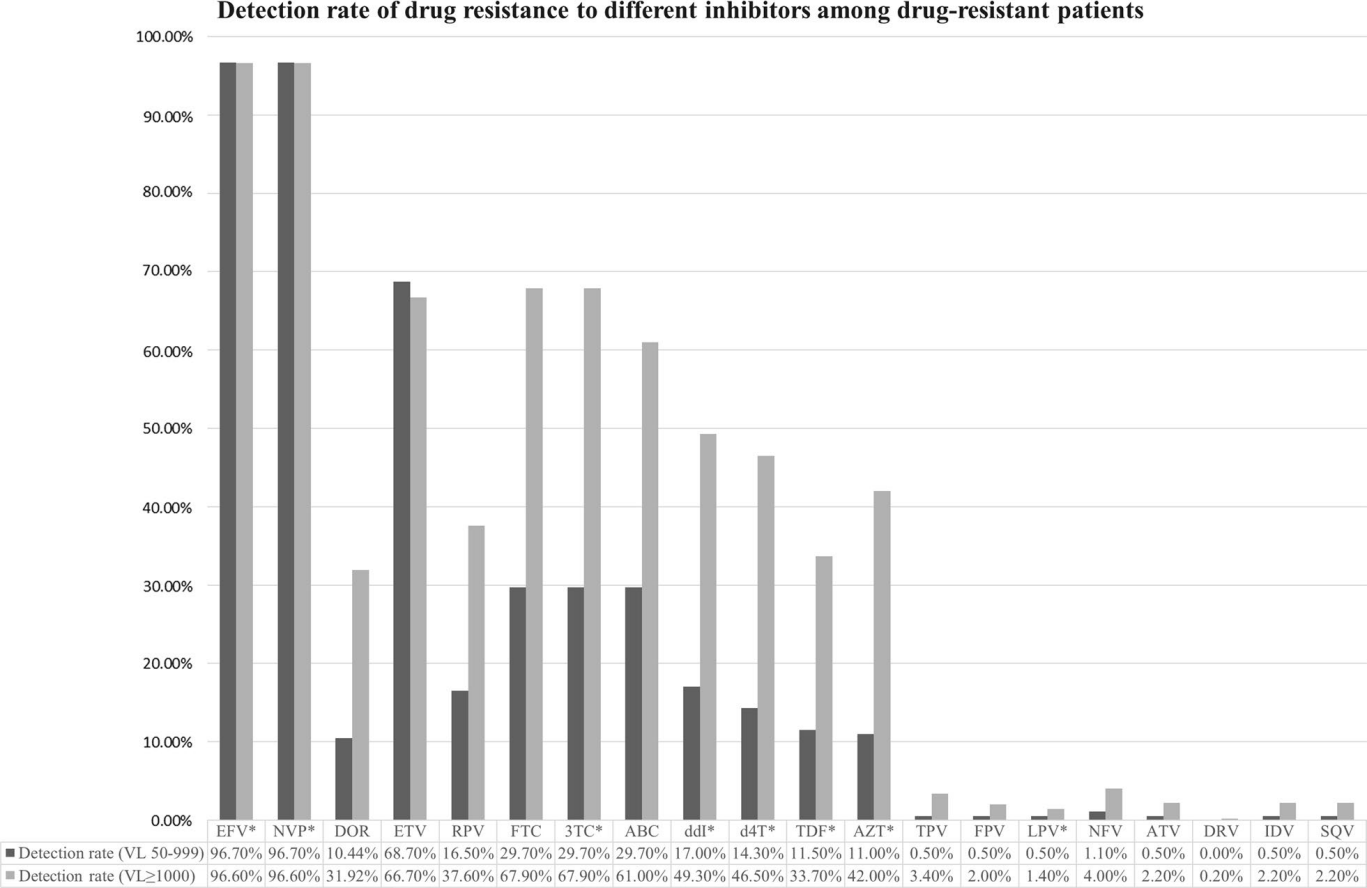
Detection rate of drug resistance to different inhibitors among drug-resistant patients who initiated ART from 2008 to 2015. EFV = Efavirenz; NVP = Nevirapine; DOR = Doravirine; FTC = Emtricitabine; 3TC = Lamivudine; ABC = Abacavir; DDI = Didanosine; D4T = Stavudine; TDF = Tenofovir; AZT = Azidothymidine; TPV = Tipranavir; LPV = Lopinavir; NFV = Nelfinavir; FPV = Fosamprenavir; ATV = Atazanavir; DRV = Darunavir; IDV = Indinavir; SQV = Saquinavir. *Provided through the National Free Antiretroviral Treatment Program (NFATP)

Of 1818 patients with VL 50–999 copies/mL, 182 (10.0%) had resistance to any type of HIV drugs. The detection rate of resistance to NNRTI drugs was higher than that to NRTI drugs and PI drugs. Of 925 patients with VF (VL ≥ 1000 copies/mL), 495 (53.5%) had resistance to any types of HIV drugs. The detection rate of resistance to NNRTI drugs was higher than that to NRTI drugs and PI drugs. Compared with patients with VL 50–999 copies/mL, the detection rate of NRTIs and PIs increased significantly in patients with VF (p < 0.01); however, the detection rate of NNRTIs did not have the same result in patients with VF (p = 0.97) (Fig. 2).

**Fig. 3:**
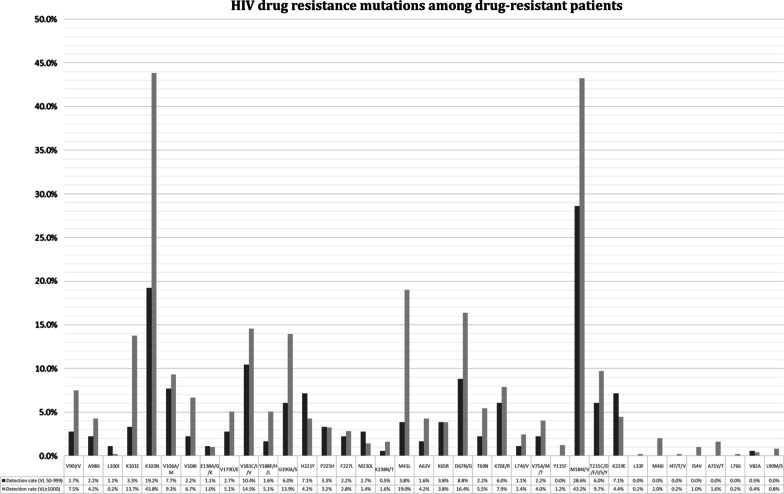
HIV drug resistance mutations among drug-resistant patients who initiated ART from 2008 to 2015

The prevalence of resistance mutations to NNRTIs, NRTIs and PIs is shown in Fig. 3. The most frequent NNRTI resistance mutations were K103N and Y181C/I/V. The most frequent NRTI resistance mutations were M184I/V. NRTI resistance mutations selected by thymidine analogues (M41L, D67N/G, K70E/R, T215C/D/F/I/S/Y and K219E) were the most frequent. The only PI mutation detected in patients with LLV was V82A (Fig. 3).

### The correlation between VL 50–200 and 201–999 copies/mL, DR and CD4 cell counts < 200 cells/*μL*

Table 2 shows the results of logistic regression models describing the relationship between LLV, DR and CD4 cell counts < 200 cells/μL. Model 1: After adjusting sex, age, occupation, marital status, route of HIV infection, duration of ART, initial ART regimens, ART regimens at survey, and missed doses, patients with VL 50–999 copies/mL and DR (AOR 3.8, 95% CI 2.6–5.5, p < 0.01), VF and no DR (3.1, 2.4–4.1, p < 0.01), VF and DR (5.8, 4.5–7.4, p < 0.01) had higher detection rates of CD4 cell counts < 200 cells/*μL*, compared with VL 50–999 copies/mL and no DR. On the other hand, patients with VL < 50 copies/mL had the lowest detection rate of CD4 cell counts < 200 cells/*μL* (0.5, 0.4–0.6, p < 0.01). Model 2: After adjusting sex, age, occupation, marital status, route of HIV infection, duration of ART, initial ART regimens, ART regimens at survey, and missed doses, patients with VL 201–999 copies/mL and DR, VF and no DR, VF and DR had higher detection rates of CD4 cell counts < 200 cells/*μL*, compared with VL 50–200 copies/mL and no DR (Table 2).

**Table 2:** The relationship between LLV, DR and CD4 cell counts < 200 cells/μL among patients who initiated ART from 2008 to 2015

Factors	N	CD4 < 200 (%)		OR (95% CI)	p	AOR (95%CI)	p
Total	6530	837	12.8%				
Model 1							
VL 50–999 and No DR	1636	195	11.9%	1.0		1.0	
VL 50–999 and DR	182	58	31.9%	3.5 (2.4, 4.9)	< 0.01	3.8 (2.6, 5.5)	< 0.01
VL < 50	3787	244	6.4%	0.5 (0.4, 0.6)	< 0.01	0.5 (0.4, 0.6)	< 0.01
VL ≥ 1000 and No DR	430	130	30.2%	3.2 (2.5, 4.1)	< 0.01	3.1 (2.4, 4.1)	< 0.01
VL ≥ 1000 and DR	495	210	42.4%	5.4 (4.3, 6.9)	< 0.01	5.8 (4.5, 7.4)	< 0.01
Model 2							
VL 50–200 and No DR	537	38	7.1%	1.0		1.0	
VL 201–999 and No DR	1099	157	14.3%	2.2 (1.5, 3.2)	0.14	2.4 (1.6, 3.6)	0.34
VL 50–200 and DR	23	2	8.7%	1.3 (0.3, 5.5)	0.23	1.2 (0.3, 5.6)	0.21
VL 201–999 and DR	159	56	35.2%	7.1 (4.5, 11.3)	< 0.01	8.0 (4.9, 13.1)	< 0.01
VL < 50	3787	244	6.4%	0.9 (0.6, 1.3)	< 0.01	0.9 (0.6, 1.2)	< 0.01
VL ≥ 1000 and No DR	430	130	30.2%	5.7 (3.9, 8.4)	< 0.01	5.7 (3.8, 8.5)	< 0.01
VL ≥ 1000 and DR	495	210	42.4%	9.7 (6.7, 14.1)	< 0.01	10.7 (7.2, 15.9)	< 0.01

CD4 cell counts < 200 cells/μL, CD4 cell counts < 200 cells/*μL*; OR, odds ratio; CI, confidence interval; AOR, adjusted odds ratio; Adjusted for: age, ethnicity, education, sex, marital status, occupation, route of HIV transmission, duration of ART, and ART regimens

## Discussion

In this large-scale Chinese multicenter analysis, patients with VL 50–999 copies/mL occurred frequently (27.8%) and increased the risk of CD4 cell counts < 200 cells/μL. Additionally, patients were more likely to experience subsequent CD4 cell counts < 200 cells/μL if they had emergent drug resistance at the time of VL 50–999 copies/mL. To be noted, accumulation of DRAM increases when maintaining a failing ARV drug regimen with VL ≥ 50 copies/mL, leading to a loss of future therapeutic options together with a large cross-resistance between ARV drug within each ARV class.

The definitions of VF and LLV vary in different periods of each country. Substantial differences exist between guidelines in developed countries, which use VL thresholds of 50–200 copies/mL to define VF, and WHO guidelines for developing countries, which apply a more lenient threshold of 1000 copies/mL [1, 6–8]. Clinical interventions are initiated upon detection of VL > 50 copies/mL in developed countries where frequent VL monitoring is performed, and the LLV is considered in the range of 50–200 copie/mL [33]. It should be noted that the definition of LLV remains 50–999 copies/mL in China, because most commercial resistance assays can only be performed on samples with VL above a minimum of 500–1000 copies/mL [19, 27, 34, 35]. We conducted a systematic nationwide survey of HIVDR in patients with VL 50–999 copies/mL in China, and compared the results with those with VL ≥ 1000 copies/mL. Among 6530 patients, 58.0% patients achieved VL less than 50 copies/mL, 27.8% had VL 50–999 copies/mL, 14.2% had a VL ≥ 1000 copies/mL and 10.4% had HIVDR. The occurrence of LLV in this study was substantially higher than that in reports from developed countries [36–38]. The occurrence of HIVDR in this study, which was higher than previously published data from China (HIVDR genotyping was performed in samples with VL ≥ 1000 copies/mL), was also higher than that reported in developed countries [11, 22, 24, 38–41]. The observed differences in prevalence of LLV and HIVDR might therefore reflect lower overall rates of virological suppression in China. Although not applicable to all patients, a tendency towards higher rates of LLV were detected in patients with poor adherence to ART. To be noted, among the 182 patients with LLV and HIVDR, 82 (45.1%) reported having missed doses in the past month; compared with 609 (16.1%) in 3,787 patients with VL < 50 copies/mL, this suggested that poor adherence would continue to be a significant problem. Therefore, we recommend that NFATP guidelines demonstrate LLV as a remarkable warning signal to have necessary clinical action, including intensification of adherence counselling and HIVDR testing.

Our results indicate that resistance testing of samples with VL < 1000 copies/mL provides clinically relevant information. It is worth noting that the prevalence of resistance to two and three ARV drugs were 26.4% and 1.1% respectively among patients with LLV. However, this prevalence increased to 72.9% and 3.6% respectively among patients with VL ≥ 1000 copies/mL. The results support guidelines recommending resistance monitoring for all patients with VL ≥ 50 copies/mL, even if genotypic resistance tests are less efficient at low viral loads [42]. Among patients with LLV and HIVDR, the proportion of drug resistance to NNRTIs, NRTIs and PIs were 96.7%, 30.2%, and 1.7%, respectively; the proportion of drug resistance to both NNRTIs and NRTIs was 27.5%. Among patients with VL ≥ 1000 copies/mL and HIVDR, the proportion of drug resistance to NNRTIs, NRTIs and PIs were 96.8%, 77.4%, and 6.1%, respectively; the proportion of drug resistance to both NNRTIs and NRTIs was 75.8%. The results showed that most of the patients with VL ≥ 1000 copies/mL and HIVDR were resistant to two or more ARV drugs, and most of those with LLV and DR were resistant to only one ARV drug. Our finding that VL between 50 and 999 copies/mL is associated with HIVDR adds to the evidence in prior studies [21, 30].

In this study, patients with HIVDR had lower CD4 counts than those without even if they had a relatively low VL. The results also showed that poor adherence (e.g. missed doses in the past month) are influenced by CD4 cell count. Poor adherence may be the reason for the low level of CD4 counts in patients with LLV (Table 1). Earlier research has shown that more than 10% of PLWH fail to achieve normalization of CD4 cell counts during ART. These patients are referred to as “inadequate immunological responders”, who show severe immunological dysfunction [43]. Previous researches had suggested that patients with CD4 cell count > 200 cells/*μL* had better recovery of immune function after ART [11, 35, 44]. Claris and Delson concluded that both VL monitoring and CD4 count monitoring play important roles in improving life expectance of patients living with HIV [45]. The level of CD4 cell count is associated with the immune system [46], and the patients on ART with poor immune function, that is, CD4 cell counts < 200 cells/μL, are more likely to develop HIVDR, indicating that PLWH with LLV and HIVDR should be detected early and change their poor adherence as soon as possible.

The results of this study showed that although patients take more time to achieve a normal CD4 cell count and less time to achieve an undetectable VL, once the CD4 cell count is normal, DRAM are reduced. The patterns of DRAM observed in this survey (Fig. 2) are consistent with previous studies [11, 22, 35, 47]. M184I/V, K103N and V181C/I/V are the common DRAM reported in China [11, 35, 39]. In this study, M184I/V was the most common DRAM detected in patients with LLV, with a prevalence of 28.6%. It is worth noting that M184I/V is mainly selected by FTC or 3TC, which is one of the important backbones in the ART regimens of NFATP [48–51]. K103N was the second most common DRAM (19.2%) detected in this study and usually selected by EFV or NVP, which are the most widely used NNRTI in NFATP [11, 35, 39]. Previous research from China has shown that the reductions in virological failure and drug resistance were strongly associated with the standardized use of TDF- or AZT-based regimens in place of the D4T-based regimen [20]. The results of this study showed that the most common DRAM detected in patients with LLV were against NNRTIs, which were used widely in NFATP [52–54]. These findings indicated that LLV episodes below 1000 copies/mL while receiving ART was associated with emerging DRAM for all ARV drug classes, suggesting earlier DRAM monitoring and ART optimization, which is identical with other results of study [55].

Our study has several limitations. First, amplification of both RT and protease genes was successful in 79.8% and 97.5% of samples with VL of 50–999 and ≥ 1000 copies/mL. This difference might lead to bias. However, multivariable analysis showed that only VL independently predicted amplification failure (p < 0.01). Exposure to the different ARV drugs was well balanced between two groups in which reverse transcriptase and protease gene amplification was successful and unsuccessful (p = 0.41). Second, only patients alive at the time of the surveys were included in this study, so the statistical relationships among LLV, CD4 cell counts < 200 cells/μL and HIVDR might be reduced. Third, the lower frequency of VL monitoring used in NFATP might in part underlie the detection rates of LLV and HIVDR.

## Conclusions

This study was intended to better understand the consequences of LLV on patients from NFATP. Our analyses showed that the risk of CD4 cell counts < 200 cells/μL was higher after LLV, especially when drug resistance occurred, and DRAM appeared as soon as LLV persistence. It included the largest patient data on this topic so far, covering 6,530 patients from various regions of China. Compared with previous studies from developed countries, the detected rates of LLV in China were higher and the risk of CD4 cell counts < 200 cells/μL and HIVDR after LLV was more pronounced, indicating that LLV is a serious threat to NFATP programmes in China.

## Data Availability

The datasets generated and/or analysed during the current study are not publicly available due to data constraint requirements from China CDC but are available from the corresponding author on reasonable request.

## Abbreviations

NFATP: National Free Antiretroviral Treatment Program
ART: Antiretroviral therapy
AIDS: Acquired Immune Deficiency Syndrome
HIV: Human immunodeficiency virus
HIVDR: HIV drug resistance
LLV: Low-level viremia
VF: Virological failure
VL: Viral load
DRAM: Drug resistance associated mutations
NNRTI: Non-nucleoside reverse transcriptase inhibitor
NRTI: Transcriptase inhibitor
PI: Protease inhibitor
